# Linear regression analysis of driving straightness among older adult drivers with and without elevated amyloid burden

**DOI:** 10.1002/alz70857_103521

**Published:** 2025-12-25

**Authors:** Anu M Kumar, Yi Lu Murphy, Amanda Cook Maher, Carol C Persad, Robert A. Koeppe, Bruno Giordani

**Affiliations:** ^1^ University of Michigan, Ann Arbor, MI, USA; ^2^ University of Michigan ‐ Dearborn, Dearborn, MI, USA

## Abstract

**Background:**

Early indications of cognitive decline and Alzheimer's disease (AD) may include minor shifts in performance on cognitively challenging, complex tasks, such as driving, which can manifest as slight difficulties in maintaining precision and control of a vehicle. Variability in straightness of driving may serve as an early indicator of cognitive change. This study evaluates the straightness of driving in older adults with and without elevated brain amyloid (Aβ) burden, a biological marker of AD.

**Method:**

Vehicular data was collected during fixed course driving trips of 20 Aβ‐positive and 20 Aβ‐negative consensus‐diagnosed cognitively normal participants (age≥65). PET centiloid scale was used to determine Aβ positivity. Vehicular data was segmented by road name, applying a linear regression model to global positioning system heading data in three‐second moving windows to predict headings and calculate deviations (absolute residuals) from the predicted path. The mean of all absolute residuals (MAR) over all windows, representing the average deviation from the predicted path, was computed. MAR values below the threshold indicate “relatively straight” driving, otherwise “deviated”. Independent sample *t*‐tests examined MAR and speed differences between the two groups on each road.

**Result:**

For the majority of roads on the fixed course, Aβ‐negative drivers exhibited a lower MAR, demonstrating more consistent driving precision. Specifically, the MAR of “relatively straight” driving on two of the longer roads (one city road and one subdivision road) was consistently higher among Aβ‐positive drivers compared to Aβ‐negative (*p*'s <0.05), indicating greater deviation and difficulty in maintaining a steady, straight driving path compared to Aβ‐negative. Additionally, the speed of “relatively straight” driving on the same city road was higher among Aβ‐negative drivers (*p*'s <0.05).

**Conclusion:**

Driving straightness in cognitively normal Aβ‐positive older adults varies significantly, with these drivers exhibiting greater fluctuations in driving precision as indicated by higher MAR results compared to Aβ‐negative drivers. These findings suggest that Aβ‐positive drivers have more difficulty maintaining a straight path and achieving higher speeds, particularly on longer road stretches, compared to Aβ‐negative peers. Further research with a larger cohort and more diverse road conditions will be undertaken to better understand the impact of amyloid burden on driving precision.

